# The importance of patients in conflict of interest declarations

**DOI:** 10.3389/fmed.2024.1365067

**Published:** 2024-03-20

**Authors:** Arch G. Mainous, Marc J. Struelens, Shisan Bao

**Affiliations:** ^1^Department of Health Services Research Management, and Policy, University of Florida, Gainesville, FL, United States; ^2^Department of Community Health and Family Medicine, University of Florida, Gainesville, FL, United States; ^3^Emeritus, Faculty of Medicine, Université libre de Bruxelles, Brussels, Belgium; ^4^Center for Laboratory and Simulation Training, School of Public Health, Center for Evidence-Based Medicine, Gansu University of Chinese Medicine, Lanzhou, Gansu, China

**Keywords:** conflict of interest, clinical trials, patient protection, institutional review board (IRB), ethics

## Introduction

It is likely that many authors and investigators wince at the thought of declaring conflicts of interest in scientific papers. Questions will be swirling through their heads like “What counts as a conflict?” and “Is that funding from a pharmaceutical company relevant to this study and needs to be declared?” They may seem to be simply one more bit of bureaucracy that slows down the dissemination of the research. Although the pharmaceutical industry supports funding of medical research, that funding is important to investigators, medical schools and hospitals thereby creating an incentive to make funders happy. Realizing that, it is important to remember that the declaration of conflicts of interest has at its primary base the safety of patients and the integrity of science. It has become apparent in articles recently submitted to *Frontiers in Medicine*, of the need to request that the authors provide additional information on issues that directly relate to the transparency in conflict of interest declarations. This editorial was authored by three Specialty Chief Editors of *Frontiers in Medicine*.

## Why do we care about conflicts of interest?

These questions emphasize the importance of transparency as it relates to the protections of patient safety and the independence of science. Are investigators acting in the best interests of the patients or are they benefitting financially, either directly or in the promotion of their career? In the past research subjects have died in clinical studies and investigators have falsified data which brought needed scrutiny on assuring research data integrity and protecting participants (1, 2). Transparency of conflicts of interest is directly linked to this concept and contributes to ensuring the public trust in the ethics of medical science.

Some patient populations have been exploited in the past in research and that has led to a reasonable distrust of medical research (3). The Trust in Medical Researchers Scale was created to assess the potential mistrust patients and in particular, minority populations may have (4). A question in the scale exemplifies this belief that researchers are acting in their interests and not in the best interests of the patient, “Researchers are more interested in helping their careers than in learning about health and disease” (4).

Acting in the interest of the investigator instead of the patient could mean that investigators have financial gain from the study that is dependent on enrolling patients and showing that a treatment works. Some investigators are paid directly for each patient that they enroll in a study (5). The patients are seen as a conduit to financial success that may take precedence in the behavior of the investigator rather than focusing on the potential risk for the patient or the advancement of science. It seems reasonable that a patient would like to know if they are contributing to a direct financial benefit to the investigator rather than helping the more noble cause of advancing science. A second way that the study may be in the investigator's financial interest and potentially not in the best interest of the patient or science could be in research that directly impact corporate interests to which the investigators benefit. Investigators affiliated with companies may have additional incentives to having study results support the use of a drug. Insider trading laws include charges of trading based on material nonpublic information about a clinical drug trial conducted to obtain Food and Drug Administration (FDA) approval to market a new drug. In other words, it is important for companies and investigators to show a benefit of the drug. The demonstration of the success or failure of a new drug may have millions of dollars in implications. Keeping corporate interests separate from scientific issues and interpretation of results is critical to move knowledge forward and provide confidence in the conclusions of a study. Addressing these financial concerns through transparency in conflict of interest declarations is a key.

## Institutional review boards

In addition to reporting financial interests, it is important that reviewers of science recognize potential conflicts. Sometimes the “letter of the law” has been followed but the perception of a conflict may be seen when examined closely. In accordance with the Declaration of Helsinki, before conducting a clinical research study, the study protocol should be pre-approved by Institutional Review Boards (IRB) in the United States, and research Ethics committees or Ethical review boards in other countries. They all follow the same international standards of Good Clinical Practice (6). Good Clinical Practice is backed in each member country by national regulations providing for clinical trial subject's protection is safeguarded through research protocols screening by Ethics committees and competent authorities, in accordance with the EU Clinical Trial Regulation (7). In the EU, local health authorities also have some oversight for study protocols.

In the United States it is quite simple to establish an institutional review board (IRB), the committee that oversees the ethics protocols for studies (8). The new IRB says that they will follow human subject protection requirements, have written procedures, and keep minutes of the meetings. For investigators working in organizations without IRBs, they have a challenge to timely review and approval of their studies that investigators in academic institutions don't have. It may be tempting for them to just set up their own IRB.

It is critically important that if non-academic institutions set up their own IRB it retains a clear sense of independence from the organization, and particularly the financial issues related to the success, failure and even conduct of certain types of studies. The IRB says that it will meet human subject protection requirements but from the outside co-mingling of individuals in corporate positions who may want a study done to help the financial standing of the company with the supposedly independent investigators can lead to meeting the “letter of the law” but not the “intent of the law.” Confidence in the study can be directly affected by an IRB that seems to be acting for corporate financial interest rather than the protection of the patient and the appropriate science. A better solution would be to contract with an independent IRB like WCG (IRB Review | WCG (wcgclinical.com).

The European Union and the United States have provided guidance but IRBs may vary from country to country. In the context of IRB processes in China, oversight is provided by the national regulatory body, 'The National Medical Products Administration' adhering to the principles outlined in the Declaration of Helsinki (9, 10). Notably, there is a prevailing practice where IRBs are typically established at least at the county level or higher, and it is uncommon for companies to have independent IRBs. That said, IRBs still need to be part of the mechanism to assure patient protection and science independence.

## Discussion

So what do we need to do to protect patients, scientific rigor and integrate that into declarations of conflicts of interest?

1. Authors should be honest in reporting conflicts where the benefit ratio is clearly to the investigator rather than the patient and science.
2. If the IRB and investigators seem to be linked together by financial interests it doesn't matter if the “letter of the law” is followed. It is better to have the protocol be reviewed and overseen by a truly independent IRB. The perception of conflict needs to be removed.
3. To enhance the awareness of the regulatory framework among Chinese authors, “The National Medical Products Administration” (http://english.nmpa.gov.cn/) should be used as a point of reference.

## Author contributions

AM: Writing – original draft, Writing – review & editing. MS: Writing – original draft, Writing – review & editing. SB: Writing – review & editing.
